# Effect of Dipeptidyl Peptidase-4 Inhibitors vs. Metformin on Major Cardiovascular Events Using Spontaneous Reporting System and Real-World Database Study

**DOI:** 10.3390/jcm11174988

**Published:** 2022-08-25

**Authors:** Yoshihiro Noguchi, Shunsuke Yoshizawa, Tomoya Tachi, Hitomi Teramachi

**Affiliations:** 1 Laboratory of Clinical Pharmacy, Gifu Pharmaceutical University, 1-25-4, Daigakunishi, Gifu-shi 501-1196, Japan; 2 Laboratory of Community Healthcare Pharmacy, Gifu Pharmaceutical University, 1-25-4, Daigakunishi, Gifu-shi 501-1196, Japan

**Keywords:** dipeptidyl peptidase-4 inhibitors, metformin, major cardiovascular events, disproportionality analysis, real-world evidence study

## Abstract

Background: Metformin had been recommended as the first-line treatment for type 2 diabetes since 2006 because of its low cost, high efficacy, and potential to reduce cardiovascular events, and thus death. However, dipeptidyl peptidase-4 (DPP-4) inhibitors are the most commonly prescribed first-line agents for patients with type 2 diabetes in Japan. Therefore, it is necessary to clarify the effect of DPP-4 inhibitors on preventing cardiovascular events, taking into consideration the actual prescription of antidiabetic drugs in Japan. Methods: This study examined the effect of DPP-4 inhibitors on preventing cardiovascular events. The Japanese Adverse Drug Event Report (JADER) database, a spontaneous reporting system in Japan, and the Japanese Medical Data Center (JMDC) Claims Database, a Japanese health insurance claims and medical checkup database, were used for the analysis. Metformin was used as the DPP-4 inhibitor comparator. Major cardiovascular events were set as the primary endpoint. Results: In the analysis using the JADER database, a signal of major cardiovascular events was detected with DPP-4 inhibitors (IC: 0.22, 95% confidence interval: 0.03–0.40) but not with metformin. In the analysis using the JMDC Claims Database, the hazard ratio of major cardiovascular events for DPP-4 inhibitors versus metformin was 1.01 (95% CI: 0.84–1.20). Conclusions: A comprehensive analysis using two different databases in Japan, the JADER and the JMDC Claims Database, showed that DPP-4 inhibitors, which are widely used in Japan, have a non-inferior risk of cardiovascular events compared to metformin, which is used as the first-line drug in the United States and Europe.

## 1. Introduction

Type 2 diabetes is associated with a variety of complications that not only worsen an individual’s quality of life [1], but also place a significant burden on medical and social welfare systems [2]. Therefore, achieving and maintaining target blood glucose levels is essential in the treatment of diabetes. The American Diabetes Association and the European Association for the Study of Diabetes [3,4] have recommended metformin as the first-line treatment for type 2 diabetes since 2006 because of its low cost, high efficacy, and potential to reduce cardiovascular events, and thus death [5]. Several studies have demonstrated the effect of metformin on reducing cardiovascular complications [6,7,8,9].

However, not only do the genetic backgrounds of diabetes onset differ between Japanese and European people [10], but Japanese type 2 diabetics are also less susceptible to insulin secretion disorders, less obese, and have a lower prevalence of cardiovascular complications than type 2 diabetics in Europe and North America [11,12]. Therefore, the Japanese Diabetes Society recommends considering the differences in the pathogenesis of type 2 diabetes between Japanese and European people, and antidiabetic therapeutic agents should be selected according to the pathogenesis of the disease, rather than using specific drugs such as metformin as a first-line treatment [13].

Several studies have reported on the trends of antidiabetic drugs in Japan and compared their efficacy and cost [14,15]. An analysis of the National Database of Health Insurance Claims and Specific Health Checkups of Japan reported that, unlike in the United States and Europe, dipeptidyl peptidase-4 (DPP-4) inhibitors are the most frequently prescribed first-line drugs for patients with type 2 diabetes in Japan [15]. Furthermore, randomized placebo-controlled cardiovascular outcome trials on DPP-4 inhibitors including the SAVOR-TIMI 53 trial (where DPP-4 inhibition with saxagliptin did not increase or decrease the rate of ischemic events) [16], the TECOS trial (where adding sitagliptin to a patient’s usual care did not appear to increase the risk of major adverse cardiovascular events) [17], the EXAMINE trial (where the rates of major adverse cardiovascular events were not increased with alogliptin as compared with a placebo) [18], and the CARMELINA trial [19] (where linagliptin being added to a patient’s usual care compared with a placebo being added to a patient’s usual care resulted in a non-inferior risk of composite major cardiovascular events) yielded non-inferior results. The CAROLINA study, a randomized controlled trial on linagliptin and glimepiride, also reported that the risk of cardiovascular events with DPP-4 inhibitors was non-inferior [20]. Meanwhile, the analyses of the Food and Drug Administration (FDA) and the Adverse Event Reporting System (FAERS), a spontaneous reporting system, suggested a mildly increased risk of cardiovascular events associated with the use of DPP-4 inhibitors [21,22]. Recently, with the increasingly widespread use of DPP-4 inhibitors, uncertainty regarding the risk of cardiovascular events associated with DPP-4 inhibitors has received increased attention given their clinical significance.

In this study, the effect of DPP-4 inhibitors on the prevention of cardiovascular events in consideration of the actual prescription of antidiabetic drugs in Japan was investigated. The Japanese Adverse Drug Event Report (JADER) database, a spontaneous reporting system in Japan, and the JMDC Claims Database, a database of health insurance claims and medical examinations in Japan, were used to conduct the analyses.

## 2. Materials and Methods

### 2.1. Disproportionality Analysis

#### 2.1.1. Data Source

This study used 565,454 registered cases from the JADER database, which was released in October 2019. Generally, only cases of reported AEs, and not of all patients using the drug, are registered in the spontaneous reporting system. Because of this, it was not possible to calculate the incidence of an AE in the JADER case data. Therefore, signal detection using disproportionality analysis was performed.

#### 2.1.2. Definitions of Suspected Drugs and Adverse Events

The suspect drugs were metformin and DPP-4 inhibitors (alogliptin, anagliptin, linagliptin, omarigliptin, saxagliptin, sitagliptin, teneligliptin, trelagliptin, and vildagliptin). However, combination drugs were excluded. The preferred term (PT) used in the Medical Terminology for Regulatory Activities/Japanese version (MedDRA/J) was used to identify adverse events, and PTs related to major cardiovascular events (defined as myocardial infarction and a stroke) and heart failure were included in this study.

#### 2.1.3. Statistical Analysis

Although several algorithms were available for signal detection [23], in this study, the Bayesian Confidence Propagation Neural Network (BCPNN), which is also used by the World Health Organization-Uppsala Monitoring Center (WHO-UMC), was used [24]. The signal indicator is the information component (IC), and the detection criterion is the lower limit of the 95% credible interval of the IC; IC_025_ > 0 (Appendix A: Table A1, Equations (A1)–(A4)).

### 2.2. Real-World Evidence Study

#### 2.2.1. Data Source

This study used the Japanese Medical Data Center (JMDC) Claims Database (JMDC Inc., Tokyo, Japan), which contains anonymous data provided by the employer’s health insurance organizations. The subscriber information, which included sex, date of birth, and diagnoses, was recorded based on International Classification of Diseases, Tenth Edition (ICD-10) codes.

#### 2.2.2. Patients

**Figure 1:**
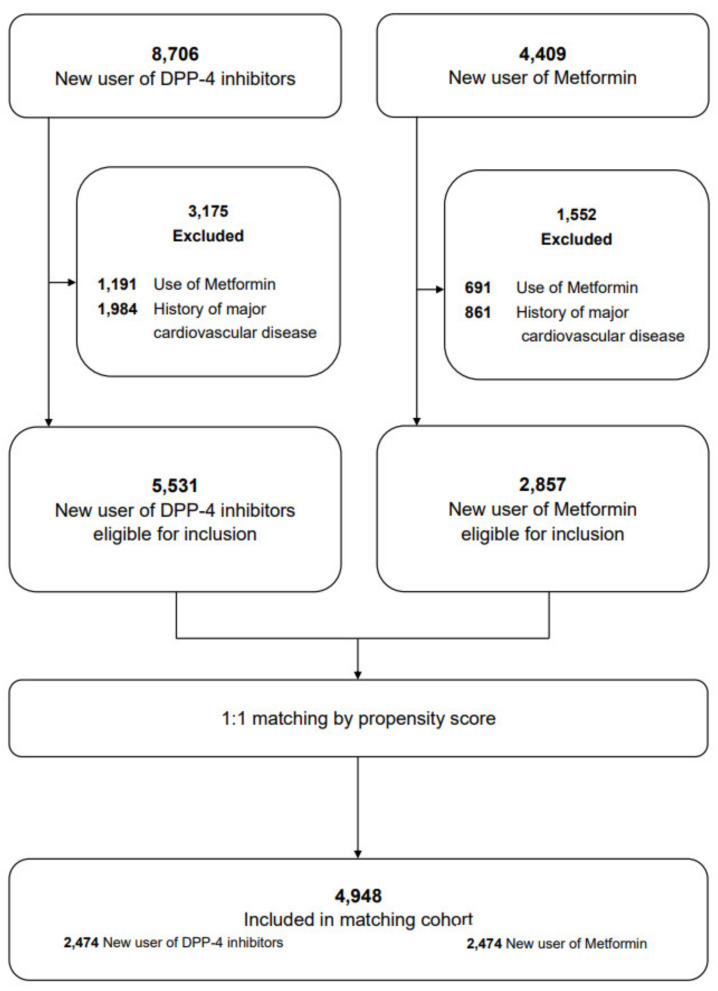
Flowchart of patient inclusion in the study cohort of the new user of dipeptidyl Peptidase-4 (DPP-4) inhibitors and metformin.

Patients aged <75 years who were new users of DPP-4 inhibitors or metformin from April 2017 to June 2017 were included in the study. New use was defined as the initiation of any drug by a patient who had not used any drug in the previous 3 months. The exclusion criteria (Table S1) were patients who had a major cardiovascular event within the past 3 months and those who had taken a controlled drug within 12 months of the start of the study (Figure 1).

#### 2.2.3. Confounder Control and Matching

The propensity score method controlled 42 potential confounders, including demographic factors, comorbidities, and drug treatment (Table S2). Propensity scores were estimated using logistic regression analysis. They were then matched in a 1:1 ratio using the nearest neighbor matching algorithm (caliper is 0.2 times the standard deviation of the logit score). 

#### 2.2.4. Outcomes

Major cardiovascular events (defined as myocardial infarction and a stroke) and heart failure were set as the endpoints of the study. Cardiovascular event outcomes were identified from the primary diagnosis assigned at admission (ICD-10 codes in Table S3), as captured by the JMDC Claims Database.

#### 2.2.5. Statistical Analysis

Patients were followed from the start of medication to the outcome event, unless they migrated, died, or reached 75 years of age, or until the study’s termination. The primary aim was to establish the non-inferiority of DPP-4 inhibitors compared with metformin over time for each cardiovascular event, defined by the upper limit of the 2-sided 95% confidence interval (CI) for the hazard ratio (HR) of DPP-4 inhibitors relative to metformin being less than 1.3 [25]. This margin (i.e., an upper limit of the 2-sided 95% CI < 1.3) was the same as in previous studies of DPP-4 inhibitors versus a placebo [15] and was deemed able to demonstrate a reassuring point estimate of the overall cardiovascular risk between study groups in the context of a non-inferiority assessment by the FDA [16]. Cox proportional hazards regression was used from the start of the treatment and the time axis was used to estimate the HR. Statistical analysis was performed using IBM SPSS Statistics 24.0 J (Armonk, New York, NY, USA).

## 3. Results

### 3.1. Disproportionality Analysis

The signals of disproportionate reporting (SDRs) for DPP-4 inhibitors were detected in major cardiovascular events (IC: 0.22, 95% credible interval: 0.03–0.40), and in the AEs of myocardial infarction (IC: 1.21, 95% credible interval: 0.87–1.55), and heart failure (IC: 0.40, 95% credible interval: 0.17–0.63). In contrast, an SDR was detected only in the AE of myocardial infarction (IC: 0.73, 95% credible interval: 0.004–1.46) with metformin treatment (Table 1).

### 3.2. Real-World Evidence Study

#### 3.2.1. Cohort

During the study period, 5531 new DPP-4 inhibitor users and 2857 new metformin users were identified (Table S4), among which a cohort of 2474 DPP-4 inhibitor users and 2474 metformin users were discovered using 1:1 propensity score matching (Table S5). The cohort was balanced for all measured covariates, with a mean age of 52.2 ± 9.74 years (mean ± standard deviation) and containing 3499 males (70.3%). The overall follow-up period was 33.1 ± 11.3 months, 32.6 ± 11.7 months in the DPP-4 inhibitor group and 33.6 ± 10.9 months in the metformin group.

#### 3.2.2. Outcomes

During the follow-up period, 239 (9.7%) DPP-4 inhibitor users and 244 (9.9%) metformin users developed major cardiovascular events. Additionally, 288 (11.6%) DPP-4 inhibitor users and 270 (10.9%) metformin users developed heart failure. Other myocardial infarctions occurred in 99 (4.0%) DPP-4 inhibitor users and in 114 (4.6%) metformin users. Moreover, strokes occurred in 161 (6.5%) DPP-4 inhibitor users and in 149 (6.0%) metformin users (Table 2).

**Figure 2:**
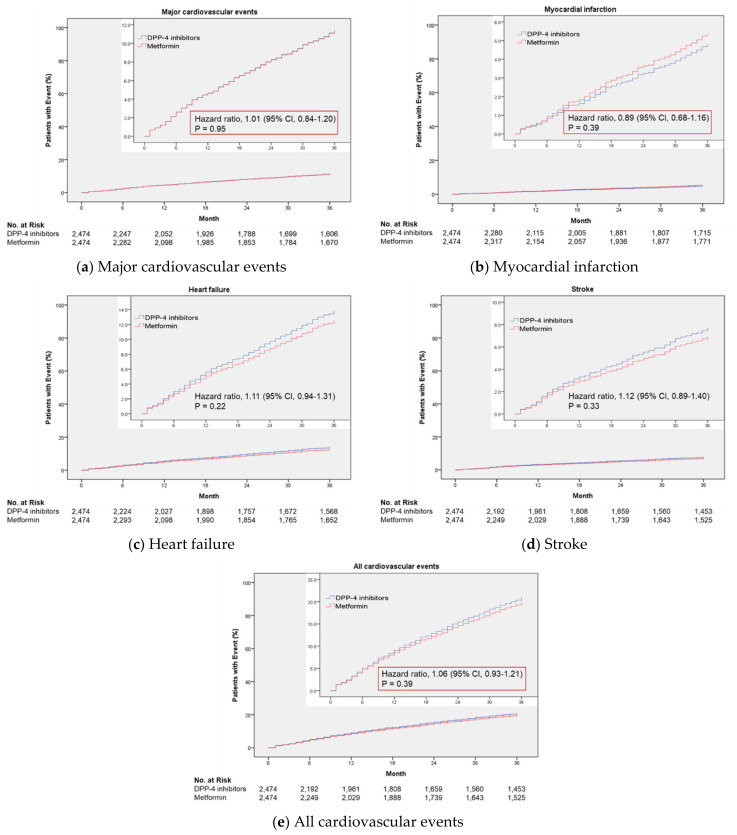
Cumulative incidence of cardiovascular events associated with use of dipeptidyl Peptidase-4 (DPP-4) inhibitors, compared with use of metformin.

The cumulative incidence of cardiovascular events is shown in Figure 2.

**Figure 3:**
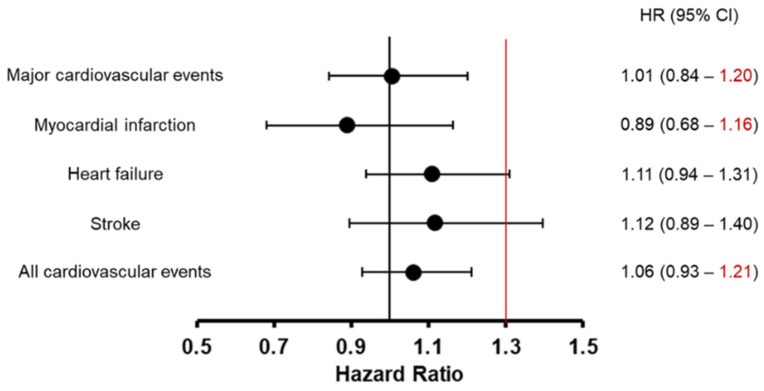
The hazard ratio of dipeptidyl Peptidase-4 (DPP-4) inhibitor use vs. metformin use.

The HRs for DPP-4 inhibitors vs. metformin were 1.01 (95% CI: 0.84–1.20) for major cardiovascular events, 0.89 (95% CI: 0.68–1.16) for myocardial infarction, 1.11 (95% CI: 0.94–1.31) for heart failure, 1.12 (95% CI: 0.89–1.40) for a stroke, and 1.06 (95% CI: 0.93–1.21) for all cardiovascular events (Figure 3).

## 4. Discussion

DPP-4 inhibitors are the most commonly prescribed first-line drugs for patients with type 2 diabetes in Japan. The prescribing pattern for type 2 diabetes in Japan differs from the pattern in the United States and in Europe. Therefore, this study investigated the effect of DPP-4 inhibitors on the prevention of cardiovascular events in Japanese patients using two different databases: the JADER, a spontaneous reporting system in Japan, and the JMDC Claims Database, a database of health insurance claims and medical examinations. 

The first part of this study examined the association of DPP-4 inhibitors and metformin with cardiovascular events using the JADER. No SDR was detected for metformin with any cardiovascular events except for myocardial infarction. In contrast, SDRs were detected with DPP-4 inhibitors in all cardiovascular events except for a stroke. These results are similar to those of previous studies that used the FAERS [21], although the definition of cardiovascular events differs between this study and previous ones. However, since the publication of several clinical trials, including the SAVOR-TIMI trial [12,13,14,15,16], clinicians have reported episodes of cardiovascular events in patients taking DPP-4 inhibitors, a situation known as “stimulated reporting” [18]. This situation can also occur naturally in Japan.

The second part of the research was a non-inferiority study of DPP-4 inhibitors and metformin on the occurrence of cardiovascular events using real-world Japanese data from the JMDC Claims Database. The results demonstrate non-inferiority in main cardiovascular events, myocardial infarction, and all cardiovascular events for DPP-4 inhibitors versus metformin. This study reaffirms clinical recommendations for antidiabetic drugs in Japan.

Several meta-analyses designed to assess the risk of major adverse cardiovascular events in type 2 diabetic patients treated with DPP-4 inhibitors have been conducted. These results suggest that DPP-4 inhibitors reduce the risk of major adverse cardiovascular events compared to diabetes drugs (with the exception of SGLT2 inhibitors which can also be used to treat chronic heart failure) and placebo [26,27,28]. Some observational studies using the Taiwan National Health Insurance Research Database [29] and the IBM MarketScan Commercial Claims and Encounters Database [30,31] also showed similar results. Thus, our results regarding the risk of cardiovascular events, taking into account antidiabetic prescribing status in Japan, are generally consistent with the results of previous studies.

There are several possible reasons for the extremely high rate of DPP-4 inhibitor prescriptions in Japan despite the availability of metformin, which is less expensive and non-inferior in cardiovascular risk [15]. The genetic background of diabetes development differs between Japanese and European people [10], and Japanese patients with type 2 diabetes are less likely to have impaired insulin secretion and are less likely to be obese than Europeans [11,12]. DPP-4 inhibitors can lower glycated hemoglobin (HbA1C) levels more effectively in Japanese people than in Europeans [11,32]. Furthermore, the risk of severe hypoglycemia is extremely low when DPP-4 inhibitors are administered alone [15]. Torii et al. reported that the risk of intensified treatment for untreated type 2 diabetes patients and the risk of developing levels of HbA1C > 7% in these patients were lower with DPP-4 inhibitors than with biguanides [33]. Furthermore, the JDS attaches great importance to the safety of antidiabetic drugs, and the proper use of biguanides and SGLT2 inhibitors is noted in its recommendations, including consideration for the elderly [15,34]. Therefore, the information revealed in this study regarding the non-inferiority of DPP-4 inhibitors to metformin with respect to cardiovascular risk may have important implications for the use of DPP-4 inhibitors in Japan.

This study had several strengths and limitations. Of the two databases used in this study, the JADER is based on spontaneous reports. This study using the JADER also has limitations similar to studies using other spontaneous reporting systems [23]. However, most of those reporting to the JADER were physicians (77.3%), followed by pharmacists (6.3%), and then lawyers (less than 0.01%) [23]. There are fewer reports from patients; therefore, the accuracy of the information in the JADER database may be higher than that of other spontaneous reporting systems.

The total number of patients using the drug cannot be obtained using the JADER database; therefore, the incidence rate cannot be calculated. Furthermore, registered cases are affected by various reporting biases [23]. This is because both physicians and patients tend not to report all cases, resulting in an underestimation of events [23].

On the contrary, the JMDC Claims Database is based on health insurance claims and medical examinations and is one of the real-world datasets in Japan. Therefore, incidence rates and HRs that cannot be calculated by the JADER can be calculated by the JMDC Claims Database. However, the JMDC Claims Database uses health insurance societies as data sources, which covers private sector employees and their families, and thus has an overwhelmingly small number of elderly people (those aged 75 and older are not included because they are part of the late-stage elderly healthcare system) [35]. Furthermore, because this study is based on secondary use of claims data, the accuracy of diagnosis depends on the accuracy of the database records, which may affect internal validity. In calculating propensity scores from the database to adjust for confounders, this study followed previous studies, but may not have included all important confounders. For example, body mass index and smoking status are important confounders of cardiovascular events but could not be considered in this study. However, analysis of these two different types of databases revealed that the risk of major cardiovascular disease with DPP-4 inhibitors, while undeniable, does not differ from the risk associated with metformin.

As a class, DPP-4 inhibitors are not associated with any increase or reduction in major cardiovascular events, all-cause mortality, or heart failure. Saxagliptin seems to be associated with an increased risk of hospitalization for heart failure [36]. Future studies will need to investigate the differential effects of individual DPP-4 inhibitors on cardiovascular events.

## 5. Conclusions

A comprehensive analysis using two different databases in Japan, the JADER and the JMDC Claims Database, showed that DPP-4 inhibitors, which are widely used in Japan, have a non-inferior risk of cardiovascular events compared to metformin, which is used as the first-line drug in the United States and Europe.

## Figures and Tables

**Table 1:** Number and signal score of adverse events.

Adverse Event	Drug Class/Drug	N_11_	IC (95% Credible Interval)
Major cardiovascular events	DPP-4 inhibitors	249	0.22 (0.03–0.40)
	Metformin	40	−0.53 (−0.98–−0.07)
Myocardial infarction	DPP-4 inhibitors	162	1.21 (0.87–1.55)
	Metformin	19	0.73 (0.004–1.46)
Heart failure	DPP-4 inhibitors	75	0.40 (0.17–0.63)
	Metformin	15	−0.78 (−1.43–−0.13)
Stroke	DPP-4 inhibitors	174	−0.07 (−0.30–0.15)
	Metformin	25	−0.96 (−1.53–−0.39)

DPP-4: dipeptidyl peptidase-4, IC: information component, N_11_: number of reports (refer to Appendix A; Table A1).

**Table 2:** Number and incidence of adverse events.

Adverse Event	DPP-4 Inhibitors (%) Total: *n* = 2424	Metformin (%) Total: *n* = 2424
Major cardiovascular events	239 (9.7)	244 (9.9)
Myocardial infarction	99 (4.0)	114 (4.6)
Heart failure	288 (11.6)	270 (10.9)
Stroke	161 (6.5)	149 (6.0)

DPP-4: dipeptidyl peptidase-4.

## Data Availability

Of the two datasets used in this study, the JADER is not permitted to be shared. However, the raw data of JADER (in Japanese only) can be accessed directly here: http://www.info.pmda.go.jp/fukusayoudb/CsvDownload.jsp (accessed on 1 August 2022). The JMDC Claims Database is available from JMDC, but is used under license in this study and is not available to the public.

## Supplementary Materials

The following supporting information can be downloaded at: https://www.mdpi.com/article/10.3390/jcm11174988/s1, Table S1: Exclusion criteria.; Table S2: Variables included in propensity score, with definitions.; Table S3: ICD-10 codes for outcome definitions., Table S4: Baseline characteristics of new users of SGLT2 inhibitors and DPP-4 inhibitors matched by propensity score (before matching).; Table S5: Baseline characteristics of new users of SGLT2 inhibitors and DPP-4 inhibitors matched by propensity score (after matching).

## Appendix A

The IC and 95% credible interval (IC_025_) are calculated using Table A1 and Equations (A1)–(A4).

**Table A1:** The 2 × 2 contingency table for signal detection.

	Target AE	Other AEs	Total
Target drug	*N*_11_	*N*_10_	*N*_1+_
Other drugs	*N*_01_	*N*_00_	*N*_0+_
Total	*N*_+1_	*N*_+0_	*N*_++_

*N*: the number of reports; AE: adverse event.

(A1)
$$E\left(IC_{11}\right)=log_{2}\frac{\left(N_{11}+\gamma_{11}\right)\left(N_{++}+\alpha \right)\left(N_{++}+\beta \right)}{\left(N_{++}+\gamma \right)\left(N_{1+}+\alpha_{1}\right)\left(N_{+1}+\beta_{1}\right)}\;$$

(A2)
$$V\left(IC_{11}\right)={\left(\frac{1}{log2}\right)}^{2}\left[\frac{N_{++}-N_{11}+\gamma -\gamma_{11}}{\left(N_{11}+\gamma_{11}\right)\left(1+N_{++}+\gamma \right)}+\frac{N_{++}-N_{1+}+\alpha -\alpha_{1}}{\left(N_{1+}+\alpha_{1}\right)\left(1+N_{++}+\alpha \right)}+\frac{N_{++}-N_{+1}+\beta -\beta_{1}}{\left(N_{+1}+\beta_{1}\right)\left(1+N_{++}+\beta \right)}\right]$$

(A3)
$$\gamma =\gamma_{11}\frac{\left(N_{++}+\alpha \right)\left(N_{++}+\beta \right)}{\left(N_{1+}+\alpha_{1}\right)\left(N_{+1}+\beta_{1}\right)},\;\gamma_{11}=1,\;\alpha_{1}=\beta_{1}=1,\;\alpha =\beta =2\;$$

(A4)
$$IC\;\left(95\%\;\mathrm{confidence}\;\mathrm{interval}\right)=E\left(IC_{11}\pm 2\sqrt{V\left(IC_{11}\right)}\right)\;\;$$
