# Supplementary material for: Effect of Dipeptidyl Peptidase-4 Inhibitors vs. Metformin on Major Cardiovascular Events Using Spontaneous Reporting System and Real-World Database Study
Source: J Clin Med. 2022 Aug 25;11(17):4988. doi: 10.3390/jcm11174988 (PMC9456525; doi:10.3390/jcm11174988)
Supplement: Supplementary file 1 [file jcm-11-04988-s001.zip › Table S1.pdf]

Table S1. Exclusion criteria

Exclusion criterion	Codes
	ICD-10
Major cardiovascular events	I00.x-I99.x, R57.0, R96.0, R96.1 (patient registers and cause of death)
Heart failure	I11.0, I13.0, I13.2, I50.x, J81.x (patient registers and cause of death)
Myocardial infarction	I21.x, I22.x (patient registers and cause of death)
Stroke	I60.x, I61.x, I63.x, I64.x (patient registers and cause of death)
	ATC code
Metformin (Taking DPP-4 inhibitors)	A10BA02, A10BD02, A10BD03, A10BD05, A10BD14, A10BD15, A10BD16, A10BD17, A10BD18, A10BD20, A10BD23, A10BD26
DPP-4 inhibitors (Taking Metformin)	A10BD19, A10BH01, A10BH02, A10BH03, A10BH04, A10BH05, A10BH06, A10BH07, A10BH08, A10BH51, A10BH52
