# Supplementary material for: Effect of Dipeptidyl Peptidase-4 Inhibitors vs. Metformin on Major Cardiovascular Events Using Spontaneous Reporting System and Real-World Database Study
Source: J Clin Med. 2022 Aug 25;11(17):4988. doi: 10.3390/jcm11174988 (PMC9456525; doi:10.3390/jcm11174988)
Supplement: Supplementary file 1 [file jcm-11-04988-s001.zip › Table S2.pdf]

Table S2. Variables included in propensity score, with definitions.

Variable	Codes
**Demographic and socioeconomic characteristics**	
Sex	
Age	
**Comorbidities**	***ICD-10***
Ischemic heart disease	I11.x (not I11.0), I20.x, (not I20.0), I24.x, I25.x
Valve disoders	I34.x-I37.x
Cerebrovascular disease	G45.x (not G45.4), G46.x, I65.x-I69.x
Atrial fibrillation	I48.x
Other arrhythmia	I44.x-I47.x, I49.x
COPD	J44.x
Other lung disease	E66.2, J27.x, J40.x-J43.x, J45.x-J47.x, J60.x-J70.x, J84.x, J92.x, J96.x, J98.2, J98.3, R09.2, Z99.x
Vemous thromboembolism	I26.x, I80.x (not I80.0), I81.x, I82.0, I82.2 - I82.9
Cancer	C00.x-C97.x (not C44.x)
Liver disease	B18.x, I85.0, I85.9, I98.2, K70.x-K77.x
Rheumatic disease	M05.x-M09.x, M30.x.-M34.x, M35.1, M35.3, M45.x
Psychiatric disorder	F04.x-F10.x, F20.x-F99.x
Fracture	M48.4, M48.5, M84.3, S02.x (not S02.5), S12.x, S22.x, S32.x, S42.x, S52.x, S62.x, S72.x, S82.x, S92.x, T02.x, T08.x, T10.x, T12.x
Arterial disease (including amputation)	E11.5, E13.5, E14.5, I65.x, I70.x, I72.x-I74.x, I77.x, K55.0, K55.1
Renal disease	E11.2, E13.2, E14.2, I12.x, I13.x, N00.x-N08.x, N17.x-N19.x, N25.x-N27.x
Diabetic complications	E11.0, E11.1, E11.3, E11.4, E11.6-E11.8, E13.0, E13.1, E13.3, E13.4, E13.6-E13.8, E14.0, E14.1, E14.3, E14.4, E14.6-E14.8, E16.0-E16.2, G59.0, G63.2, G99.0, H28.0, H35.8, H36.0, L98.4, M14.2, M14.6, M90.8
**Diabetes drugs**	*ATCcode*
SGLT2	A10BK01, A10BK02, A10BK03 ,A10BK04 ,A10BK05, A10BK06 ,A10BK07
Sulphonylureas	A10BB, A10BD01, A10BD04, A10BD06
Insulin	A10AB, A10AC, A10AD, A10AE

GLP1 receptor agonists	A10BJ01, A10BJ02, A10BJ03, A10BJ05
Other antidiabetics (glitazones, glinides, acarbose)	A10BG, A10BX, A10BD04, A10BD06, A10BD09, A10BF01
**Other drugs in the past year**	
ARB/ACE-I	C09A-C09D
Calcium-channel blocker	C08C, C08D
Loop diuretic	C03C, C03EB
Other diuretic	C03A, C03B, C03D, C03EA
Beta-blocker	C07
Digoxin	C01AA05
Nitrate	C01DA
Platelet inhibitors	B01AC
Anticoagulant	B01AA, B01AF, B01AE07, B01AX05
Lipid lowering drug	C10
Antidepressant	N06A
Antipsychotic	N05A
Anxiolytic, hypnotic, or sedative	N05B, N05C
Beta-2 agonist inhalant	R03AC
Anticholinergic inhalant	R03BB
Glucocorticoid inhalant	R03BA, R03AK
Oral glucocorticoid	H02AB
NSAID	M01A
Opioid	N02A
