# Supplementary material for: Effect of Dipeptidyl Peptidase-4 Inhibitors vs. Metformin on Major Cardiovascular Events Using Spontaneous Reporting System and Real-World Database Study
Source: J Clin Med. 2022 Aug 25;11(17):4988. doi: 10.3390/jcm11174988 (PMC9456525; doi:10.3390/jcm11174988)
Supplement: Supplementary file 1 [file jcm-11-04988-s001.zip › Table S3.pdf]

Table S3. ICD-10 codes for outcome definitions.

**Outcome**	**ICD-10 codes**
Major cardiovascular events	I21.x, I22.x, I60.x, I61.x, I63.x, I64.x (patient registers)
Heart failure	I11.0, I13.0, I13.2, I50.x, J81.x (patient registers and cause of death)
Myocardial infarction	I21.x, I22.x (patient registers and cause of death)
Stroke	I60.x, I61.x, I63.x, I64.x (patient registers and cause of death)
Cardiovascular death	I00.x-I99.x, R57.0, R96.0, R96.1 (patient registers and cause of death)
