# Supplementary material for: Effect of Dipeptidyl Peptidase-4 Inhibitors vs. Metformin on Major Cardiovascular Events Using Spontaneous Reporting System and Real-World Database Study
Source: J Clin Med. 2022 Aug 25;11(17):4988. doi: 10.3390/jcm11174988 (PMC9456525; doi:10.3390/jcm11174988)
Supplement: Supplementary file 1 [file jcm-11-04988-s001.zip › Table S4.pdf]

Table S4. Baseline characteristics of new users of SGLT2 inhibitors and DPP4 inhibitors matched by propensity score (before matching)

Characteristics	All patients (n =8,388)	DPP-4 inhibitors (n =5,531)	Metformin (n =2,857)	*P*
Male sex	5,964 (71.1)	3,981 (72.0)	1,983 (69.4)	0.015
Age (mean ± standard deviation)	53.3 ± 9.88	55.5 ± 9.26	51.0 ± 10.4	< 0.001
**Comorbidities**				
Ischemic heart disease	635 (7.6)	462 (8.4)	173 (6.1)	< 0.001
Valve disoders	55 (0.7)	41 (0.7)	14 (0.5)	0.200
Cerebrovascular disease	140 (1.7)	102 (1.8)	38 (1.3)	0.088
Atrial fibrillation	80 (1.0)	63 (1.1)	17 (0.6)	0.017
Other arrhythmia	310 (3.7)	226 (4.1)	84 (2.9)	0.009
COPD	44 (0.5)	32 (0.6)	12 (0.4)	0.426
Other lung disease	1,006 (12.0)	690 (12.5)	316 (11.1)	0.060
Vemous thromboembolism	83 (1.0)	59 (1.1)	24 (0.8)	0.353
Cancer	1,082 (12.9)	776 (14.0)	306 (10.7)	< 0.001
Liver disease	2,810 (33.5)	1,882 (34.0)	928 (32.5)	0.157
Rheumatic disease	214 (2.6)	159 (2.9)	55 (1.9)	0.008
Psychiatric disorder	777 (9.3)	495 (8.9)	282 (9.9)	0.177
Fracture	133 (1.6)	93 (1.7)	40 (1.4)	0.357
Arterial disease (including amputation)	65 (0.8)	48 (0.9)	17 (0.6)	0.191
Renal disease	1,777 (21.2)	1,095 (19.8)	682 (23.9)	< 0.001
Diabetic complications	1,488 (17.7)	853 (15.4)	635 (22.2)	< 0.001
**Use of diabetes drug**				
SGLT2	1,292 (15.4)	673 (12.2)	619 (21.7)	< 0.001
Sulphonylureas	1,382 (16.5)	978 (17.7)	404 (14.1)	< 0.001
Insulin	849 (10.1)	421 (7.6)	428 (15.0)	< 0.001
GLP1 receptor agonists	295 (3.5)	14 (0.3)	281 (9.8)	< 0.001
Other antidiabetics (glitazones, glinides, acarbose)	1,607 (19.2)	1,070 (19.3)	537 (18.8)	0.558
**Use other drugs**				
ARB/ACE-I	3,032 (36.1)	2,034 (36.8)	998 (34.9)	0.098
Calcium-channel blocker	2,752 (32.8)	1,909 (34.5)	843 (29.5)	< 0.001
Loop diuretic	72 (0.9)	64 (1.2)	8 (0.3)	< 0.001
Other diuretic	232 (2.8)	157 (2.8)	75 (2.6)	0.623
Beta-blocker	442 (5.3)	304 (5.5)	138 (4.8)	0.216
Digoxin	6 (0.1)	4 (0.1)	2 (0.1)	1.000
Nitrate	46 (0.5)	32 (0.6)	14 (0.5)	0.644
Platelet inhibitors	355 (4.2)	247 (4.5)	108 (3.8)	0.152
Anticoagulant	71 (0.8)	52 (0.9)	19 (0.7)	0.210
Lipid lowering drug	3,780 (45.1)	2,537 (45.9)	1,243 (43.5)	0.042
Antidepressant	362 (4.3)	223 (4.0)	139 (4.9)	0.079
Antipsychotic	136 (1.6)	84 (1.5)	52 (1.8)	0.316
Anxiolytic, hypnotic, or sedative	860 (10.3)	596 (10.8)	264 (9.2)	0.030
Beta-2 agonist inhalant	71 (0.8)	48 (0.9)	23 (0.8)	0.803
Anticholinergic inhalant	20 (0.2)	16 (0.3)	4 (0.1)	0.240
Glucocorticoid inhalant	205 (2.4)	127 (2.3)	78 (2.7)	0.233
Oral glucocorticoid	222 (2.6)	169 (3.1)	53 (1.9)	0.001
NSAID	1,204 (14.4)	819 (14.8)	385 (13.5)	0.101
Opioid	11 (0.1)	10 (0.2)	1 (0.0)	0.112
