# Supplementary material for: Effect of Dipeptidyl Peptidase-4 Inhibitors vs. Metformin on Major Cardiovascular Events Using Spontaneous Reporting System and Real-World Database Study
Source: J Clin Med. 2022 Aug 25;11(17):4988. doi: 10.3390/jcm11174988 (PMC9456525; doi:10.3390/jcm11174988)
Supplement: Supplementary file 1 [file jcm-11-04988-s001.zip › Table S5.pdf]

Table S5. Baseline characteristics of new users of SGLT2 inhibitors and DPP4 inhibitors matched by propensity score (after matching)

	All patients	DPP-4 inhibitors	Metformin	
**Characteristics**	**（n =4,948）**	**（n =2,474）**	**（n =2,474）**	***P***
Male sex	3,499 (70.7)	1,758 (71.1)	1,741 (70.4)	0.617
Age (mean ± standard deviation)	52.2 ± 9.74	52.4 ± 9.89	52.0 ± 9.57	0.209
**Comorbidities**				
Ischemic heart disease	310 (6.3)	164 (6.6)	146 (5.9)	0.319
Valve disoders	26 (0.5)	12 (0.5)	14 (0.6)	0.845
Cerebrovascular disease	71 (1.4)	37 (1.5)	34 (1.4)	0.811
Atrial fibrillation	36 (0.7)	20 (0.8)	16 (0.6)	0.616
Other arrhythmia	152 (3.1)	76 (3.1)	76 (3.1)	1.000
COPD	28 (0.6)	17 (0.7)	11 (0.4)	0.344
Other lung disease	530 (10.7)	259 (10.5)	271 (11.0)	0.613
Vemous thromboembolism	49 (1.0)	27 (1.1)	22 (0.9)	0.478
Cancer	579 (11.7)	310 (12.5)	269 (10.9)	0.077
Liver disease	1,612 (32.6)	820 (33.1)	792 (32.0)	0.413
Rheumatic disease	91 (1.8)	44 (1.8)	47 (1.9)	0.833
Psychiatric disorder	449 (9.1)	223 (9.0)	226 (9.1)	0.921
Fracture	69 (1.4)	31 (1.3)	38 (1.5)	0.467
Arterial disease (including amputation)	32 (0.6)	18 (0.7)	14 (0.6)	0.595
Renal disease	1,086 (21.9)	541 (21.9)	545 (22.0)	0.918
Diabetic complications	935 (18.9)	461 (18.6)	474 (19.2)	0.663
**Use of diabetes drug**				
SGLT2	893 (18.0)	449 (18.1)	444 (17.9)	0.853
Sulphonylureas	697 (14.1)	366 (14.8)	331 (13.4)	0.165
Insulin	534 (10.8)	266 (10.8)	268 (10.8)	0.963
GLP1 receptor agonists	27 (0.5)	14 (0.6)	13 (0.5)	1.000
Other antidiabetics (glitazones, glinides, acarbose)	911 (18.4)	465 (18.8)	446 (18.0)	0.486
**Use other drugs**				
ARB/ACE-I	1,712 (34.6)	861 (34.8)	851 (34.4)	0.788
Calcium-channel blocker	1,530 (30.9)	785 (31.7)	745 (30.1)	0.230
Loop diuretic	20 (0.4)	12 (0.5)	8 (0.3)	0.503
Other diuretic	133 (2.7)	68 (2.7)	65 (2.6)	0.861
Beta-blocker	246 (5.0)	128 (5.2)	118 (4.8)	0.556
Digoxin	4 (0.1)	2 (0.1)	2 (0.1)	1.000
Nitrate	21 (0.4)	11 (0.4)	10 (0.4)	1.000
Platelet inhibitors	188 (3.8)	97 (3.9)	91 (3.7)	0.710
Anticoagulant	36 (0.7)	18 (0.7)	18 (0.7)	1.000
Lipid lowering drug	2,129 (43.0)	1,069 (43.2)	1,060 (42.8)	0.818
Antidepressant	217 (4.4)	107 (4.3)	110 (4.4)	0.890
Antipsychotic	77 (1.6)	37 (1.5)	40 (1.6)	0.818
Anxiolytic, hypnotic, or sedative	462 (9.3)	236 (9.5)	226 (9.1)	0.660
Beta-2 agonist inhalant	42 (0.8)	21 (0.8)	21 (0.8)	1.000
Anticholinergic inhalant	10 (0.2)	6 (0.2)	4 (0.2)	0.754
Glucocorticoid inhalant	129 (2.6)	63 (2.5)	66 (2.7)	0.858
Oral glucocorticoid	101 (2.0)	51 (2.1)	50 (2.0)	1.000
NSAID	648 (13.1)	311 (12.6)	337 (13.6)	0.292
Opioid	1 (0.0)	0 (0.0)	1 (0.0)	1.000
